# The Many Faces of JAKs and STATs Within the COVID-19 Storm

**DOI:** 10.3389/fimmu.2021.690477

**Published:** 2021-07-13

**Authors:** Alice H. Grant, Armando Estrada, Yoshira M. Ayala-Marin, America Y. Alvidrez-Camacho, Georgialina Rodriguez, Elisa Robles-Escajeda, Denisse A. Cadena-Medina, Alejandro C. Rodriguez, Robert A. Kirken

**Affiliations:** Department of Biological Sciences, The University of Texas at El Paso, El Paso, TX, United States

**Keywords:** JAK, STAT, cytokine, cytokine storm, cytokine release syndrome, JAK inhibitor, COVID-19, SARS-CoV-2

## Abstract

The positive-sense single stranded RNA virus, Severe Acute Respiratory Syndrome Coronavirus 2 (SARS-CoV-2), resulted in a global pandemic with horrendous health and economic consequences not seen in a century. At a finer scale, immunologically, many of these devastating effects by SARS-CoV-2 can be traced to a “cytokine storm” resulting in the simultaneous activation of Janus Kinases (JAKs) and Signal Transducers and Activators of Transcription (STAT) proteins downstream of the many cytokine receptor families triggered by elevated cytokines found in Coronavirus Disease 2019 (COVID-19). In this report, cytokines found in the storm are discussed in relation to the JAK-STAT pathway in response to SARS-CoV-2 and the lessons learned from RNA viruses and previous Coronaviruses (CoVs). Therapeutic strategies to counteract the SARS-CoV-2 mediated storm are discussed with an emphasis on cell signaling and JAK inhibition.

## Introduction

### Covid-19 Pathogenesis and the Cytokine Storm

Late 2019 a positive single strand RNA virus crossed over to humans, causing a Coronavirus (CoV) related pneumonia in Wuhan China. The pathogen responsible for the following CoV Disease 2019 (COVID-19) global pandemic was identified as Severe Acute Respiratory Syndrome Coronavirus-2 (SARS-CoV-2) on January 7^th^, 2020 (1). Unlike past CoVs which can be mildly pathogenic others like SARS-CoV and Middle East Respiratory Syndrome CoV (MERS-CoV) can result in severe disease and fatality (2). SARS-CoV-2 falls into the latter, with an estimated 178,837,204 infected and greater than 3,880,450 deaths worldwide as of June 23^rd^, 2021 (World Health Organization). For many the cause of death is due to Acute Respiratory Distress Syndrome (ARDS)/respiratory failure, septic shock, or multiorgan system dysfunction (Centers for Disease Control and Prevention). Additionally, COVID-19 related strokes are on the rise likely due to thromboembolism complications (3, 4).

Many of these overt symptoms result from a cytokine release syndrome or “cytokine storm”, with uncontrolled anti- and pro-inflammatory components reaching beyond the local site of infection and resulting in systemic collateral damage (5). Disproportional outcomes of COVID-19 pathology are largely attributed to this dysfunctional immune response (6, 7). This notion is supported by findings that asymptomatic individuals display less inflammatory cytokine profiles and a subtle immune response (8).

**Figure 1:**
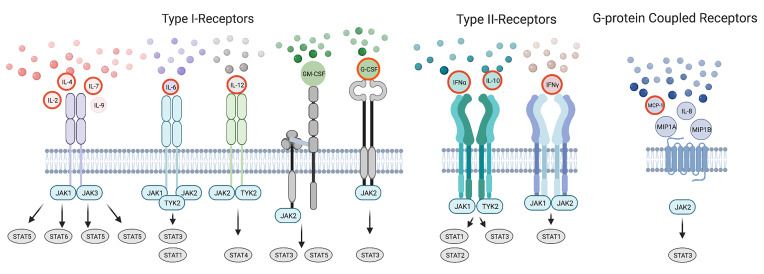
The Cytokine Storm in COVID-19 Utilizes JAKs and STATs. A simple schematic showing elevated cytokines in COVID-19 signaling by Type I, Type II, Immunoglobulin, and G-protein coupled receptors with those correlated to disease severity outlined in red. Paired JAKs are seen signaling immediately downstream of receptors directly activating STATs (solid line).Only the predominantly activated STAT is depicted for shown cytokines.

SARS-CoV-2 infected, hospitalized patients display elevated levels of Interleukins (ILs) IL-2, IL-4, IL-7, IL-9, IL-6, Granulocyte-Colony Stimulating Factor (G-CSF), Granulocyte Macrophage-CSF (GM-CSF), Interferon α2 (IFNα2), Interferon γ (IFNγ), IL-10, IL-1α, IL-1Ra, IL-1β, Macrophage-CSF (M-CSF), IL-12, Tumor Necrosis Factor α (TNFα), IL-17, IL-8, Macrophage Inflammatory Proteins 1A (MIP1A), Macrophage Inflammatory Proteins 1B (MIP1B), Monocyte Chemoattractant Protein-1 (MCP-1), IFNγ-Inducible Protein 10 (IP-10), Fibroblast Growth Factor (FGF), Hepatocyte Growth Factor (HGF), Vascular Endothelial Growth Factor (VEGF), and Platelet-Derived Growth Factor (PDGF) (6, 9). These elevated cytokines subsequently activate multiple cytokine receptor families belonging to the Type I, Type II, Immunoglobulin Superfamily, G-Protein Coupled, TNFα and Growth Factor Receptors. Many of these cytokine receptors rely on the Janus Kinases (JAKs) and Signal Transducers and Activators of Transcription (STAT) proteins to immunologically eradicate the SARS-CoV-2 pathogen and restore immune homeostasis. Cytokines utilizing Type I, Type II and G-Protein Coupled Receptors propagate direct signals through JAKs and STATs and many have been linked to disease severity (**Figure 1**). Others are regulated by or cross-talk with JAK-STAT pathways.

## JAK And STATs

JAKs consist of seven Janus Homology (JH) domains, JH1-JH7, shared across JAK1, JAK2, JAK3 and Tyrosine Kinase 2 (TYK2) (10, 11). JH1 harbors the kinase domain involved in Tyr phosphorylation while the JH2 consists of an “inactive” pseudo-kinase domain regulating overall activity. JH3-JH7 consists of the 4.1R, Ezrin, Radixin, Moesin (FERM) and Src Homology 2 (SH2) domains involved in receptor binding. Canonically, JAKs act by transferring the γ-phosphate of ATP to the hydroxyl side chain of Tyr residues residing on paired receptors, recruiting in SH2 containing proteins, most notably the STATs. Once captured by the receptor, STATs are phosphorylated by the JAKs among other SRC family kinases (12). Subsequent disengagement from the receptor results in STAT dimerization formed by SH2 interactions with phosphorylated Tyr enhancing nuclear translocation and transcriptional activities. The seven STAT family members contain six domains that include the N-terminal, coiled-coil, DNA binding, linker, SH2, and a transactivation domains (10, 13). And although structurally similar, their target genes are overlapping but also unique (14).

## JAKS And The Immunological Response

**Figure 2:**
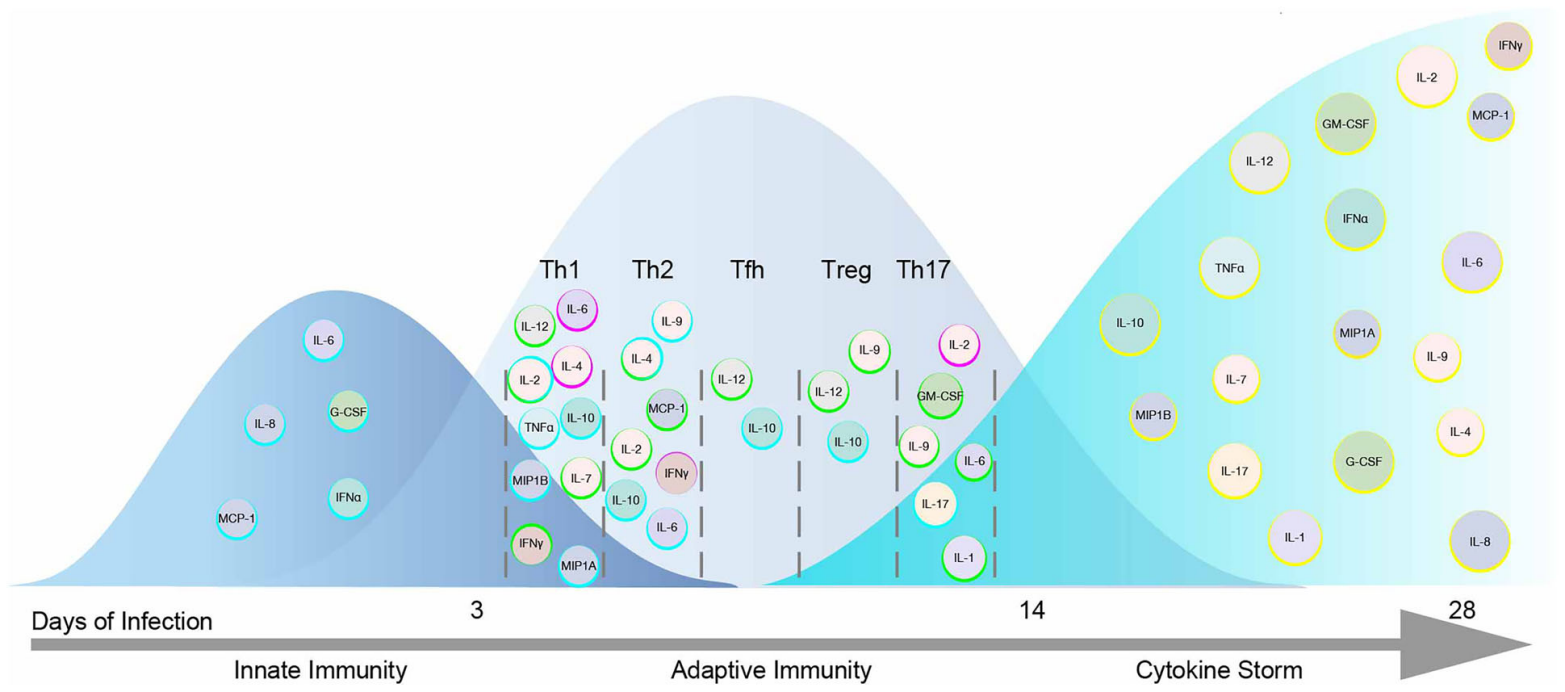
Cytokines within the Storm are Depicted in the Immunological Response to SARS-CoV-2 Infection. A model of the immunological response to SARS-CoV-2 infection displaying cytokines within the storm including those that signal by JAKs and STATs. Cytokines are placed in the immunological timeline based on their expected role in innate and adaptive responses to SARS-CoV-2 infection. Cytokines enhancing immune function are outlined in blue. Cytokines promoting Th differentiation are outlined in green while those inhibiting Th differentiation are outlined in fuchsia. Cytokines are later shown during the COVID-19 cytokine storm outlined in yellow.

The JAK-STAT pathway is essential for various stages of immunity that ranges from initiating signaling events required for innate and adaptive responses to the pathological stage of driving the storm (15, 16). For example, the innate immune system, launches an anti-viral response through JAK dependent IFNs, pro-inflammatory cytokines and chemokines including those found in the cytokine storm (IFNα2, IL-6, IL-8, G-CSF, MCP-1) (17, 18). This initial response seeks to inhibit viral progression and to activate the adaptive immune system. In this second phase, cytokines elicit an appropriate adaptive response to viral infection by facilitating CD4^+^ T helper (Th) differentiation and or CD8^+^ T cytotoxic (Tc) and B cell function. Many cytokines cross-talk and are dependent upon the JAK-STAT pathway to affect the spectrum of Th phenotypes that normally respond to a range of pathogenic variation including among others Th1, Th2, T follicular helper (Tfh), T regulatory (Treg) and Th17 cells (19, 20). Some of these canonical cytokines sway Th differentiation including IFNγ, IL-2, IL-12 towards Th1; IL-2, IL-4 to Th2; IL-12 to Tfh; IL-2 to Treg; and IL-6, IL-1 to Th17 subsets (19). IL-4 and IFNγ inhibit Th1 and Th2 responses respectively (21, 22). Others are produced by Th subtypes including IFNγ, IL-2, TNFα in Th1; IL-4, IL-6, IL-10 in Th2; IL-17 in Th17; and IL-10 in both Tfh and Treg cells. Additionally IL-7, IL-9, MIP1A, MIP1B, MCP-1, and GM-CSF can influence differentiation and adaptive immunity. Failure of the adaptive arm to clear the infection results in a cytokine storm consisting of these latter JAK signaling cytokines (**Figure 2**).

Typically, Th1 cells drive cellular immunity largely accomplished by Tc and NK cells while Th2 and Tfh gear towards humoral immunity utilizing plasma B cells. Treg cells restore and maintain immunological homeostasis (23) while Th17 are crucial in eliciting a response against extracellular pathogens. The signature Th phenotype that succeeds at clearing SARS-CoV-2 infection between asymptomatic, mild/moderate, and severe/critical cases remains elusive. It is implied that asymptomatic and mild to moderate disease achieve viral clearance by intact innate and adaptive responses while hyperinflammation leads to severe/critical disease (24).

It is likely that SARS-CoV-2 immunity in mild to moderate disease is largely accomplished by Th1 responses. Supporting this claim, roughly 70% of non-hospitalized patients that recovered from SARS-CoV-2 infection exhibit virus specific CD8^+^ Tc cells (25). Furthermore, patients that recovered from past SARS-CoV infection achieve lasting immunity through virus specific memory CD4^+^ and CD8^+^ T cells (2). Others have proposed Th2 responses in children translate to the mild outcomes of SARS-CoV-2 infection (26). Although, Tfh and Th2 responses are seen in hospitalized patients (27) with the latter response associated with severe symptoms to SARS-CoV-2 (28). Thus, it is unknown if humoral responses are sufficient to clear SARS-CoV-2 infection (29). Interestingly, unexposed SARS-CoV-2 individuals can present Th cells that recognize the spike viral protein, indicative of past exposure to other CoV (25, 30). Indeed, sera from SARS-CoV-2-uninfected donors contain antibodies against the S2 subunit of the spike protein that can neutralize SARS-CoV-2 (25). SARS-CoV-2–uninfected children and adolescents are more likely than adults to contain these cross reactive and ‘protective’ antibodies (31). These findings support that immunogenic sites of the spike protein are conserved between other CoV and SARS-CoV-2 (32).

Past CoV have revealed antagonism against the adaptive immune response by impairing CD4^+^ and CD8^+^ T cell activation (2). And comparably, severe COVID-19 patients display ineffective Th1 phenotypes indicated by decreased levels of CD8^+^ Tc cells coupled with markers of exhaustion (33). Lymphopenia in severe COVID-19 patients is met with decreases in both CD4^+^ and CD8^+^ T cells (33, 34). Th cells tend to shift towards Th17 cells in severe patients (35, 36) while Tregs are decreased in critically ill patients (35, 37). Details on how Tc and Th cells aid or worsen the progression of COVID-19 are discussed elsewhere (38). Taken together, many severe cases of COVID-19 are accompanied by an uncontrolled immunological response. The latter triggered in part by mechanisms of SARS-CoV-2 immune evasion leading to non-productive cytokine profiles (39). Each of these cytokines are discussed below with an emphasis on those that signal through the JAK-STAT pathway.

## Type I Cytokines

### γ Chain Family

The common γ chain (γc) cytokines are critical for the survival of T and B cell lineages that generate adaptive immunity to viral infections such as SARS-CoV-2. Emerging data from SARS-CoV-2 patients show increases in nearly all γc cytokines including, IL-2, IL-4, IL-7, and IL-9, were all except IL-9 are associated with disease severity (9, 40). Their respective receptors lacking intrinsic catalytic activity pair with γc and recruit JAK1 and JAK3 to phosphorylate Tyr residues drawing in STAT proteins. Their redundancy ends in part by each STAT protein that is dominantly recruited and phosphorylated in response to a given γc cytokine. Although STATs can be activated at varying degrees, IL‐2, IL‐7, and IL-9 preferentially activate STAT5A and STAT5B, while IL‐4 activates STAT6 (20).

Many γc cytokines are released by naïve or differentiated CD4^+^ T and CD8^+^ Tc cells to strengthen the adaptive immune response (41). For example, Th1 cells release IL-2 enhancing Tc differentiation and expansion (42). IL-2 and IL-4 induce Th2 differentiation, where downstream activation of STAT6 by IL-4 regulates Ig gene transcription and switch recombination in plasma B cells (20, 43). IL-7 can induce Th1 differentiation and, like IL-2, is important for the maintenance of memory T cells (44, 45). IL-9, produced by Th2 and Th17 cells has been shown to enhance the suppressive functions of natural Treg cells and promote Th17 differentiation (46, 47).

The simultaneous release of γc cytokines suggests that SARS-CoV-2 fails to generate a single Th phenotype and succeeds at eliciting immunological chaos; perhaps an attempt of redirecting away from cell mediated and humoral responses that aid in viral clearance. Alternatively, elevated levels of γc cytokines in COVID-19, may reflect an attempt to strengthen the adaptive arm of the immune system. Despite the probable increases in γc cytokines acting as growth factors for lymphocytes, lymphopenia is frequently observed in severe COVID-19 patients (33, 34). This paradox has been tackled in depth by others with various explanations (48) including T cell exhaustion. While IL-2 signals are essential for T cell expansion, prolonged IL-2 can mediate exhaustion of CD8^+^ Tc as well (49, 50).

### IL-6

IL-6 is a signature cytokine of inflammation correlating with COVID-19 mortality (51). Binding of IL-6 to its receptor promotes dimerization with gp130 leading to the activation of JAK1, JAK2 and TYK2 (52). JAKs then phosphorylate gp130 to recruit SH2 containing STAT1 and STAT3 that subsequently become phosphorylated. Within various cells, STAT1 and STAT3 form either homo or hetero-dimers acting as transcription factors to regulate expression of multiple genes (53, 54). This in part allows for the pleiotropic activities of IL-6 ranging from polarizing naïve Th cells to supporting differentiation of non-immune cells (55). For example, IL-6 promotes Th2, and Th17 differentiation while inhibiting Th1 responses (56, 57). Among many cytokines discussed hereafter IL-6 also regulates coagulation (58) likely contributing to COVID-19 thrombosis related mortality (59, 60). To ameliorate the effects of IL-6, antibodies generated against IL-6 or its receptor are under clinical trial investigation. However, preliminary data are showing limited efficacy against COVID-19 (61, 62).

### IL-12

IL-12 utilizes IL-12p40, IL-12 Receptor β1 (IL-12Rβ1) and IL-12Rβ2 bound to Tyk2 and JAK2, respectively (63). The latter chain creates docking sites for STAT4 that undergoes phosphorylation to regulate transcription and signaling. STAT4 Ser phosphorylation in response to IL-12 has been shown crucial for T cell IFNγ secretion a typical Th1 response (64). IL-12 is also important for Tfh differentiation (65). IL-12p40 mRNA increases rapidly after CNS CoV infection and contributes toward morbidity associated with viral encephalitis (66). Additionally, increased IL-12 expression is correlated with COVID-19 severity (40). Yet, IL-12 is needed by host viral defenses given its influence on Th differentiation. And vaccine development against the SARS-CoV-2 S-protein includes a component of IL-12 based therapy currently in Phase I clinical trials (clinicaltrials.gov).

### G-CSF

Severe COVID-19 patients display elevated levels of G-CSF a primary growth factor for neutrophil differentiation (40). G-CSF signaling is mediated through the Tyr receptor kinase G-CSF Receptor (G-CSFR) and βc, activating JAK2 and subsequently STAT3 (67, 68). In one study, neutrophilia occurred in more than half of severe COVID-19 patients (69) and is likely attributed in part by the actions of G-CSF.

### GM-CSF

GM-CSF also activates JAK2 and STAT3/5 through its GM-CSF Receptor α and βc subunit and is involved in Th17 differentiation (70). Known to link the CNS with inflammation it is perhaps not surprising that elevated levels of GM-CSF are seen in CNS SARS-CoV positive children exhibiting Encephalitis-like syndrome (71). Neurological symptoms seen in SARS-CoV-2 cases have yet to be distinguished as a result from encephalitis, meningitis, or secondary effects of severe infection (72). SARS-CoV-2 viral particles and RNA are observed in neuroanatomical areas that receive olfactory tract projections (73). And other routes of entry for SARS-CoV-2 neuro-invasion have been purposed (74). CNS damage continues to be observed in COVID-19 patients discussed by De Felice et al. (75) and CNS pathologies warrant further investigation.

## Type II Cytokines

### Type I and II IFN

Type I IFN (IFN-I) and Type II IFNγ are key in alerting and protecting the body against viral infections (76). Interestingly, IFNα2 and IFNγ are highly expressed in severe COVID-19 patients. IFN-I acting on their ubiquitously expressed receptors utilize JAK1 and TYK2 for signaling. Once activated the JAKs phosphorylate STAT1 and STAT2, enabling them to complex with IFN Regulatory Factor 9 (IRF9) and initiate transcription of classical IFN stimulated genes involved in antiviral response, immune regulation, and anti-proliferation (76–78). IFNγ utilizes JAK1 and JAK2 signaling to promote STAT1 antiviral activity and drive Th1 differentiation and thus cellular immunity (79, 80). Priming infected cells for destruction, many viruses antagonize IFN responses by targeting the JAK-STAT pathway (81). For example, SARS-CoV Non-Structural Protein 1 (NSP1) acts as a virulence factor for evading the IFN response in part by decreasing phosphorylation of STAT1 (82). STAT3, also downstream of IFN but mainly induced by IL-6, is found dephosphorylated at Tyr 705 in the presence of SARS-CoV (83). Similarly, SARS-CoV-2 ORF3b truncated viral protein in addition to ORF6, and ORF8 can suppress IFN-I signaling (84, 85). Cell lines infected with SARS-CoV-2 show a reduction in JAK1, JAK2, TYK2 and STAT2 protein expression. Moreover, virus-infected cells are not able to induce STAT1, STAT2 and STAT3 phosphorylation to the same extent than non-infected cells in response to IFNα2 (86).

A strategy to harness the initial actions of IFN-I and maintain balance of the immune response, active forms of IFN-I are being investigated in clinical trials against COVID-19 (87, 88). Akin to SARS-CoV, SARS-CoV-2 infection responds similarly to IFN-I therapy, where the timing of its use is critical for efficacy. Specifically, early IFN-I therapy is associated with reduced mortality, while late therapy increases mortality in a retrospective study (89). The timing of effective IFN-I administration is intuitive given its presence in the milieu of cytokines needed to trigger first phase innate immunity, while its delayed presence triggers hyperinflammation (90). However, appealing, IFN-I strategies should be taken with caution given that SARS-CoV-2 can utilize IFNα to induce ACE2 expression, its common route of entry (91).

### IL-10

The type II cytokine receptor for IL-10, signals through JAK1 and TYK2 activating STAT3, yet in contrast to IFN yields an anti-inflammatory response inhibiting Th1 differentiation (92). Treg cells achieve their anti-inflammatory effects in part by secreting IL-10 (19). Unlike MERS-CoV, elevated concentrations of IL-10, are seen in SARS-CoV-2 and may be linked to the decreased numbers in CD8^+^ Tc cells (93, 94). Certain viruses encode IL-10 homologs to suppress the immune system, thus the high levels of IL-10 seen in severe COVID-19 patients likely benefit SARS-CoV-2 (9, 40, 95). However, with the right timing IL-10 can aid in viral clearance when coupled to IFNγ (96). Lastly the anti-inflammatory effects by IL-10 can inhibit coagulation activation and stimulate fibrinolysis (97).

## G Protein-Coupled Receptors

Signaling *via* G-Protein coupled receptors, IL-8, MIP1A, MIP1B, MCP1, IP-10 are elevated in COVID-19 patients (9, 40), conceivably stimulating migration of immune cells to the site of infection. Binding of these chemokines to their respective receptors, IL-8 to CXCR1/CXCR2, MIP1A to CCR1/CCR5, MIP1B to CCR5/CCR8, MCP1 to CCR1/CCR2, and IP-10 to CXCR3 activate the JAK/STAT pathway (98–101) all recruiting JAK2 and STAT3 (98–103), except IP-10. Although IP-10 can indirectly activate downstream STAT1, STAT4 and STAT5 (104). IL-8 acts as a chemoattractant for many immune cells, dominantly recruiting neutrophils for host defense. Although recruited neutrophils are critical for clearing infections, excessive neutrophil invasion could be the culprit of lung injury observed in COVID-19 pneumonia (69). MIP1A and MIP1B, the former linked to COVID-19 severity, are associated with the trafficking of CD8^+^ and CD4^+^ T cells, respectively (105, 106). Despite primarily attracting macrophages and lymphocytes (107), MIP1A can also attract neutrophils (108). MCP-1 has been associated with recruiting both pathological macrophages/neutrophils (109) and virus clearing CD8^+^ Tc cells (110). In addition to acting as a chemoattract, MCP-1 can polarize Th2 responses (98, 111, 112). In contrast, IP-10 stimulates a Th1 response (113) recruiting primarily T cells (114) and its presence correlates with viral load (115–117). Both MCP-1 and IP-10 are linked to COVID-19 severity (9, 40).

## Regulation And Cross-Talk With JAK-STAT Pathways

### Immunoglobulin Superfamily Receptors

IL-1α, IL-1β and IL-1Ra act as damage-associated molecular pattern (DAMP) detectors (118), likely activated by the direct and collateral damage of SARS-CoV-2. Binding IL-1 (IL-1α, IL-1β) to IL-1 receptor type I (IL-1R1) results in a robust pro-inflammatory response (119). In contrast, IL-1Ra produces an anti-inflammatory response (119). Although these factors do not utilize the JAK-STAT pathway, it has been reported that IL-1 inhibits IL-6 driven STAT1 activation (120). M-CSF belongs to the immunoglobulin superfamily and does not cross-talk with the JAK-STAT pathway. Likely propagated by the heightened Th17 response, IL-17 is found at elevated levels in severe COVID-19 patients (40). IL‐17A–IL‐17F form either homo- or heterodimers and signal through IL-17 Receptor A (IL‐17RA) and IL‐17RC subunits (121) to promote inflammation. The IL‐17RA is relatively expressed in the lungs (http://www.proteinatlas.org) and its activation can promote chemoattractants for neutrophil invasion (122). There is evidence that Th17 responses are implicated in severe lung pathology and mortality induced by CoVs (123). Furthermore, it has been shown to signal in astrocytes *via* an indirect JAK2, STAT1 and STAT3 axis (124, 125).

### Tumor Necrosis Factor Receptors

While TNFα does not signal through JAK-STAT proteins, it is discussed here because it is found elevated in COVID-19 patients requiring intensive care (9). TNFα signals through two receptors, TNFR1 and TNFR2 triggering inflammatory pathways, and immune modulation respectively (126). TNFα is also released by differentiated Th1 cells. Cross-talk between TNFα and the JAK-STAT pathway has been suggested by few reports demonstrating changes in STAT3 and STAT5 following activation of TNFR1 and TNFR2 (126, 127). Prothrombotic effects of TNFα are thought to be mediated by TNFR2 rather than TNFR1 (128). Circulating TNFα, IFNγ, IL-1, IL-6, IL-8, and MCP-1 all effect tissue factor expression that initiate coagulation (129, 130). Additionally, TNFα, IL-1, IL-6, IL-12 and IL-2 can induce thrombin (130) which converts fibrinogen to fibrin (131) involved in the cross-linking that stabilizes blood clots. TNFα and IL-1 can also inactivate fibrinolysis (130). Taken together, TNFα along circulating cytokines may contribute to COVID-19 thrombotic complications.

### Growth Factor Receptors

Growth factors signal mainly through receptors containing their own intrinsic catalytic activity, bypassing the need for JAKs. Increased levels of growth factor in SARS-CoV-2 patients include VEGF, FGF, HGF and PDGF that are involved in processes such as angiogenesis, morphogenesis and fibrotic remodeling. FGF, VEGF, HGF and PDGF have all been shown to be regulated by or cross-talk with JAK-STAT pathways. For example, VEGF expression along with IL-6 can be induced by IL-17 through STAT1 to promote angiogenesis (125). JAK2 and STAT5 are utilized by FGF receptor 2 (FGFR2) to facilitate morphogenesis (132). HGF stimulates the recruitment and phosphorylation of STAT3 that is also relevant to morphogenesis (133). And PDGF can facilitate airway remodeling by cross-talk *via* a JAK2, STAT1 and STAT3 pathway (134).

## Targeting The JAK Family

**Figure 3:**
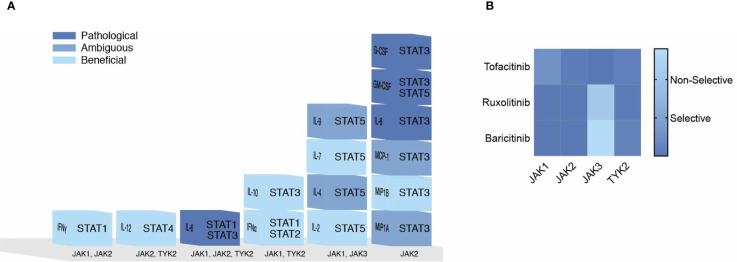
Pathogenicity of the Cytokine Storm Driven by JAKs. **(A)** COVID-19 elevated cytokines directly signaling by the JAK-STAT pathway are shown in a heat map corresponding to their predicted pathogenicity in COVID-19 based on **Table 1**. **(B)** JAK inhibitors are shown with their selectivity of each JAK based on their IC50 values.

JAKs represent a major therapeutic target for the treatment of COVID-19. However, inhibition of all JAKs might not have beneficial outcomes. Correlating COVID-19 disease severity with elevated cytokines shouldn’t imply each cytokine is pathogenic. Cytokines act upon multiple cell types and across distal and proximal sites. In this review the impact of cytokines signaling directly through JAKs were sought to predict their beneficial immunological, ambiguous or pathological responses to SARS-CoV-2 (**Table 1**), color-coded light to dark (respectively), and organized corresponding to JAKs and STATs in **Figure 3A**. IL-2, IL-7, IFNα2, IFNγ, IL-12, IL-10 and MIP1B are predicted to elicit a beneficial immune response against SARS-CoV-2. While IL-6, G-CSF, GM-CSF, and IL-8 might provoke unwanted pathological outcomes. These cytokines utilize different combinations of JAKs and STATs where more often “beneficial” cytokines recruit JAK1 and JAK3 in contrast to “pathogenic” cytokines that predominantly recruit JAK2. The latter being associated with downstream activation of STAT3, also suggested as a plausible target for the treatment of COVID-19 (135). Of note, while few STAT3 direct inhibitors are FDA approved, there are added concerns of inhibitory cross-reactivity with STAT1, critical for antiviral responses (136).

**Table 1:** Predicted actions of JAK signaling cytokines in SARS-CoV-2 infection.

Consequence	Function	Impact	Collective Consequence
IL-2	Th1 differentiation	1	Beneficial
	Th2 differentiation	0	
	Maintains memory T-cells	1	
	Treg differentiation	1	
	Inhibits Th-17	1	
	Coagulation	-1	
IL-4	Inhibits Th1 differentiation	-1	Ambiguous
	Th2 differentiation	0	
	Immunoglobulin switch	1	
IL-7	Th1 differentiation	1	Beneficial
	Maintains memory T-cells	1	
IL-9	Treg activation	1	Ambiguous
	Released by Th2 cells	0	
	Th17 differentiation	-1	
IL-6	Th2 response	0	Pathological
	Th17 differentiation	-1	
	Coagulation	-1	
IL-12	Th1 differentiation	1	Beneficial
	Tfh differentiation	0	
	T-cell IFN secretion	1	
	Involved in encephalitis	-1	
G-CSF	Neutrophilia	-1	Pathological
GM-CSF	Th17 response	-1	Pathological
	CNS inflammation	-1	
IL-8	Neutrophil recruitment	-1	Ambiguous
MIP1A	Recruits CD8 cells	1	Ambiguous
	Neutrophil recruitment	-1	
MIP1B	Recruits CD4 cells	1	Beneficial
MCP-1	Th2 responses	0	Ambiguous
	Recruits CD8 cells	1	
	Neutrophil recruitment	-1	
	Macrophage recruitment	0	
IFNα2	Antiviral response	1	Beneficial
	Immune regulation	1	
	Anti-proliferation	1	
	ACE2 expression	-1	
IFNγ	Antiviral response	1	Beneficial
	Th1 differentiation	1	
IL-10	Produced by Tregs	1	Beneficial
	Counters coagulation	1	
	Inhibits Th1 differentiation	-1	

1, Predicted to positively affect COVID-19.

0, Predicted to have no or neutral impact on COVID-19.

-1, Predicted to negatively affect COVID-19.

The use of selective JAK inhibitors for the treatment of COVID-19 have conflicting views. Prioritizing IFN antiviral/antibacterial immunity, suggest sparing JAK combinations downstream of IFN-I (JAK1/TYK2) and IFNγ (JAK1/JAK2) (15). In contrast, Schett et al. suggest targeting JAK2, downstream of IL-6 and GM-CSF to ameliorate hyperinflammation, and spare JAK1/JAK3 downstream of IFN-I, IL-2, IL-15, IL-21 and IFNγ involved in viral clearance (137). Others have proposed TYK2 as a target, given that variants effecting expression are related to COVID-19 critical illness (138).

These conflicting views on selective JAK inhibition may be resolved by considering the role of JAK dependent cytokines within the immunological timeline discussed previously (see **Figure 2**). Mild to moderate responses to SARS-CoV-2 are likely associated with intact innate and adaptive responses while severe disease progresses with hyperinflammation brought on by the cytokine storm (24). Thus, strategies to combat SARS-CoV-2 in early mild to moderate disease would benefit from sparing initial (JAK1/TYK2) and (JAK1/JAK2) combinations given IFNs role in innate immunity and transition to Th1 adaptive immunity. However, during the cytokine storm in COVID-19, targeting JAK2 and sparing JAK1/JAK3 combinations may preserve cell mediated and or humoral defenses against SARS-CoV-2. Three FDA approved JAK inhibitors, Tofacitinib, Ruxolitinib and Baricitinib can act on each JAK kinase at varying degrees (**Figure 3B**). Each has undergone clinical trials for treating COVID-19 patients (15) where drug combinations using Baricitinib have been approved by emergency use authorization (139). In agreement with the former analysis, Baricitinib preferentially targets JAK1/JAK2 while somewhat sparing JAK3 inhibition. In general, JAK blockade has shown to reduce recovery time and mortality compared to standard treatments (140).

## Discussion

Classic JAK inhibitors exploit the JH1 active site acting as ATP mimetics and because they act on more than one JAK they are casually considered to be pan-inhibitors rather than JAK specific inhibitors (141). The majority of the pipeline of JAK inhibitors are classified as Type I and Type II, depending on whether they target an active or inactive kinase, respectively. The ATP-binding site is highly conserved across kinases allowing them to act broadly. For example, JAK inhibitors are said to cross-react with AP2-associated protein kinase 1 (AAK1) that regulates endocytosis and could thus prevent SARS-CoV-2 entry (142). In contrast, Type III and Type IV inhibitors function to allosterically disrupt either proximal or distal changes to the ATP-binding site respectively (143). These distal and non-entirely conserved regions might be ideal for specific JAK inhibition.

At present, there are limited allosteric JAK inhibitors available and none of which have been approved by the FDA (144). New allosteric strategies might include, exploiting endogenous mechanisms of JAK regulation. For example, small peptide inhibitors mimicking as substrates could accomplish steric hindrance in a JAK specific manner by targeting unique substrate specific residues within the JH1. Additionally, identifying negative regulatory phosphorylation sites within JAKs could be key in determining which kinase specific phosphatases could be effectively inhibited to disrupt subsequent downstream signals. Lastly, the pseudokinase JH2 domain acts as an intrinsic regulator of JAK activity and identifying key residues, or non-conserved motifs, required for negative regulation could be exploited for JAK inhibition.

The use of JAK inhibitors for treating COVID-19 have been met with caution given their potential risk of thrombosis (145–147). Whether these concerns apply to pan-JAK inhibitors and/or specific JAK inhibitors should be addressed. Regardless, the hypercoagulability state that contributes to thrombotic effects in COVID-19 patients may be compounded by pan-JAK inhibition (145, 148). Thus, such patients experiencing complications of thrombosis may not benefit from “pan” JAK inhibitors (15). Perhaps the ability to effectively counter these complications reflects differences in the hemostatic system that differs across age and sex similar to the risk of COVID-19 pathogenesis (6, 149–151). Nevertheless, hospitalized COVID-19 patients can benefit from a recently approved treatment regimen consisting of baricitinib, in combination with remdesivir.

The FDA recommends continued efforts in drug strategies to accelerate recovery, slow disease progression and lower mortality in COVID-19. These efforts might benefit from specific JAK2 inhibitors with the rationale as provided in this review. Lastly, it should be noted that JAK inhibition is one of many treatment strategies for COVID-19 patients that are not managed by currently approved treatment strategies. Such efforts must continue during vaccine distribution programs and continued for unvaccinated individuals or when vaccination is ineffective. For now, JAK inhibitors are accessible in this time sensitive fight against SARS-CoV-2 and a path to dodge the storm.

## Author Contributions

AHG conceived, wrote, and finalized figures of the manuscript. AE contributed writing and generated figures. YA-M, AA-C, GR, and ER-E contributed to writing and editing. DC-M and ACR contributed writing. RAK conceived and critically revised the manuscript. All authors contributed to the article and approved the submitted version.

## Funding

This work was supported by Grant 5U54MD007592 from the National Institutes on Minority Health and Health Disparities (NIMHD), a component of the National Institutes of Health (NIH) and by the RISE Scholars Program at UTEP from the National Institute of General Medical Sciences (NIGMS) Grant R25GM069621-18.

## Conflict of Interest

The authors declare that the research was conducted in the absence of any commercial or financial relationships that could be construed as a potential conflict of interest.
